# Quercetin Supplementation Improves Neuromuscular Function Recovery from Muscle Damage

**DOI:** 10.3390/nu12092850

**Published:** 2020-09-17

**Authors:** Ilenia Bazzucchi, Federica Patrizio, Roberta Ceci, Guglielmo Duranti, Stefania Sabatini, Paolo Sgrò, Luigi Di Luigi, Massimo Sacchetti

**Affiliations:** 1 Laboratory of Exercise Physiology, Department of Movement, Human and Health Sciences, University of Rome “Foro Italico”, Piazza Lauro De Bosis 6, 00135 Roma, Italy; federica.patrizio@libero.it (F.P.); massimo.sacchetti@uniroma4.it (M.S.); 2 Laboratory of Biochemistry of Movement, Department of Movement, Human and Health Sciences, University of Rome “Foro Italico”, Piazza Lauro De Bosis 6, 00135 Roma, Italy; roberta.ceci@uniroma4.it (R.C.); guglielmo.duranti@uniroma4.it (G.D.); stefania.sabatini@uniroma4.it (S.S.); 3 Endocrinology Unit, Department of Movement, Human and Health Sciences, University of Rome “Foro Italico”, Piazza Lauro De Bosis 6, 00135 Roma, Italy; paolo.sgro@uniroma4.it (P.S.); luigi.diluigi@uniroma4.it (L.D.L.)

**Keywords:** muscle damage, DOMS, elbow flexors, electromyography, muscle weakness

## Abstract

This study was aimed at investigating whether quercetin (Q) may improve the recovery of neuromuscular function and biochemical parameters in the 7 days following an eccentric exercise-induced muscle damage (EEIMD). Sixteen men (25.9 ± 3.3 y) ingested Q (1000 mg/day) or placebo (PLA) for 14 days following a double-blind crossover study design. A neuromuscular (NM) test was performed pre–post, 24 h, 48 h, 72 h, 96 h and 7 days after an intense eccentric exercise. The force–velocity relationship of the elbow flexor muscles and their maximal voluntary isometric contraction (MVIC) were recorded simultaneously to the electromyographic signals (EMG). Pain, joint angle, arm circumference, plasma creatine kinase (CK) and lactate-dehydrogenase (LDH) were also assessed. The results showed that Q supplementation significantly attenuated the strength loss compared to PLA. During the recovery, force–velocity relationship and mean fibers conduction velocity (MFCV) persisted significantly less when participants consumed PLA rather than Q, especially at the highest angular velocities (*p* < 0.02). A greater increase in biomarkers of damage was also evident in PLA with respect to Q. Q supplementation for 14 days seems able to ameliorate the recovery of eccentric exercise-induced weakness, neuromuscular function impairment and biochemical parameters increase probably due to its strong anti-inflammatory and antioxidant action.

## 1. Introduction

During eccentric exercise, skeletal muscle is exposed to stretch and overload which causes structural damage to myofibers and secondary inflammation resulting from leukocyte infiltration into the damaged tissues [1]. Signs and symptoms of eccentric exercise-induced muscle damage (EEIMD) often persist for several days after conclusion of exercise and typically include muscle pain, localized swelling, temporary decrements in maximal force-generating capacity, altered joint kinematics, elevated levels of intramuscular enzymes (i.e., creatine kinase (CK) and lactate dehydrogenase (LDH)), elevations in markers of inflammation and various interleukins [2,3]. Although the specific mechanisms responsible for these signs and symptoms are not completely understood, inflammation and reactive oxygen species (ROS) are presumably among the primary causes.

Because EEIMD inevitably leads to considerable discomfort and impairs subsequent athletic performance or training quality, the alleviation of symptoms by nutritional and supplementation strategies may be advantageous to individuals who require a rapid recovery between bouts of physical activity. Unfortunately, the effectiveness of many emergent nutritional supplements with anti-inflammatory and antioxidant properties, including polyphenols, has not been fully explored.

Recent studies have shown how quercetin (Q), a flavonol-type polyphenol, could protect from muscle damage [4,5]. Q supplementation, in fact, has been shown to reduce the entity of strength loss caused by action potential propagation impairment and myofibrillar disruption which characterized an eccentric exercise-induced muscle damage. What is still unclear is whether this protective action can not only reduce the extent of muscle damage but whether it can also improve the recovery from the damage itself.

Studies on the time course of neuromuscular function symptoms after EEIMD, have reported a rather slow recovery of muscle strength [6] and of electromyographic parameters, with a complete restoration of the baseline condition after 2 weeks from the muscle damage [7]. In agreement, the primary outcome of the present study was on muscle strength to test the hypothesis that 14 days of supplementation with Q would not only prevent the strength loss induced by eccentric exercise, but would also improve the recovery of neuromuscular function in the 7 days following the damage.

## 2. Materials and Methods

### 2.1. Participants

Sixteen healthy men (age 25.9 ± 3.3 y; BMI 23.4 ± 2.0) were recruited to participate in the present study. The exclusion criteria were: (1) reported symptoms of cardiac, metabolic, neurological or renal diseases; (2) reported or evidenced presence of stress events that could interfere and/or contraindicate with the experimental protocol, procedures and evaluations; (3) reported use of any kind of dietary supplements during the period of 6 months before the protocol.

The protocol of the study was approved by the Ethics Committee of the “Sapienza” University of Rome (number 3798). Participants were informed of the experimental procedures and the possible risks of the study before providing written informed consent. All participants were engaged in low-to-moderate physical activity which was assessed with the IPAQ (International Physical Activity Questionnaires) prior to their enrolment (IPAQ: 563 ± 327 MET-min/week). All the volunteers were taking no medications, anabolic agents, any kind of dietary supplements particularly containing vitamins and/or antioxidants, and amino acids supplementation. They refrained from Q-containing foods for the whole duration of the investigation. The subjects drank water ad libitum and the breakfast was standardized and consumed 2 h before each session. Subjects were also requested to refrain from strenuous exercise or major stress events starting from 48 h before each experimental session.

### 2.2. Research Design

**Figure 1:**
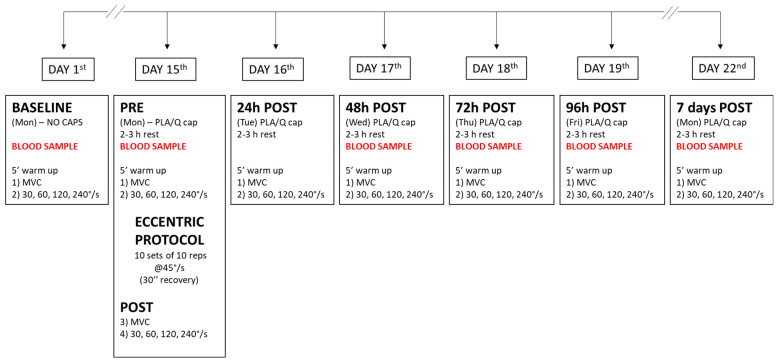
Diagram of the research design. BASELINE: baseline measurements before treatment; PRE: pre-test; MVC: maximal voluntary contraction; POST: post-test; PLA: placebo; Q: quercetin.

This study used a double-blind, placebo-controlled, crossover design with each volunteer serving as their own control. Volunteers were randomly assigned to placebo (PLA) or Q supplementation and crossed over to the other treatment after a wash-out period of 3 weeks [8]. The first visit to the laboratory was used to familiarize the subjects with the experimental measures. No blood samples were collected during this trial. Then, the whole study consisted of 7 experimental sessions which started at the same time of the day (±2 h) (see Figure 1). The order of the supplements (Q vs. PLA) was randomly assigned. The neuromuscular (NM) test was performed on the upper limb. The arm (right (R) or left (L)) exposed to the eccentric protocol was reversed when the protocol was repeated with the other treatment. The association between treatment (Q or PLA) and arm (R or L) was also randomly assigned so that the limb dominance was not uniquely associated with the supplementation with Q or PLA. Functional parameters and NM evaluation were assessed before the treatment (BASELINE), after 14 days of PLA/Q intake (PRE), after the eccentric protocol (POST), and 24 h, 48 h, 72 h, 96 h, 7 days during the recovery.

### 2.3. Treatment

A pre-intervention evaluation was carried out (DAY 1—BASELINE). Afterward, participants were randomly assigned to the first treatment (PLA or Q). They ingested 2 capsules containing 500 mg of Q aglycone in crystalline powder (Farmalabor srl, Milano, Italy) to achieve a daily dose of 1000 mg [9]. Placebo pills (500 mg of magnesium stearate + cornstarch) tasted and appeared the same of Q. Subject compliance to the treatment was checked by the investigators involved in the research project.

### 2.4. NM Evaluation

During each experimental session, a complete NM evaluation was performed. Isokinetic dynamometry (Kin-Com, Chattanooga, TN, USA) was used to assess the elbow flexion torque as already described [4]. The individual-specific adjustments to the instrument were recorded (according to anatomical differences), to guarantee an accurate re-positioning. The surface EMG signals were recorded with a linear array of 4 electrodes (silver bars 5 mm long, 1 mm thick, 10 mm apart; OTBioelettronica, Torino, Italy) from the biceps brachii muscle (BB). The optimal position and orientation of the electrodes were determined as previously described [10,11]. The EMG signals were detected in a single-differential mode. Double-differentials were computed off-line and were used for further analysis. Signals were amplified, band-pass filtered (10–450 Hz; EMG USB2+, OTBioelettronica, Torino, Italy), sampled at 2048 Hz, recorded, and stored on a personal computer.

Volunteers performed a standardized warm-up and then they started the NM test which consisted of: (1) maximal voluntary isometric contraction (MVIC); (2) force–velocity relationship (FV). In each trial, a strong verbal encouragement was given by the test leader.

(1) MVIC. The joint angle was fixed at 90° (0°, full extension). The MVIC task consisted of rapidly increasing the force exerted by elbow flexors to a maximum. All participants were verbally encouraged to exceed the target force, producing a maximal contraction “as hard as possible” and to maintain it for at least 2–3 s before relaxing. A minimum of 3 maximal attempts were performed separated by 5 min to recover from fatigue.
(2) FV test. Participants were asked to perform 3 maximal isokinetic back-to-back elbow flexions. The angular velocities were: 30, 60, 120, 240°/s with 5-min rest between the sets [12,13]. The range of motion (ROM) was 100° starting from 40° to 140°. The order of the angular velocities was randomized.

### 2.5. Eccentric Protocol

Each participant completed 10 bouts of 10 maximal lengthening contractions of the elbow flexors; each set was separated by a 30-s rest. Each eccentric contraction lasted 2 s and was followed by a 6-s rest period in which the participant relaxed while the dynamometer arm returned automatically back to 50° of elbow flexion as previously described [4]. The angular velocity was set at 45°/s and subjects were instructed to resist maximally during the entire range of motion (from 40° to 140°).

### 2.6. Indices of EEIMD

A 10-cm visual analogue scales (VAS) was used to assess pain intensity. The scale was fixed at right with “worst pain imaginable” (score of 10) at left with “no pain” (score of 0) and [14]. A goniometer was used to assess the elbow joint angle while participants stood upright with their arms relaxed. The rationale to measure the joint angle is that eccentric exercise has been demonstrated to induce a rise in passive tension of the involved muscles which could result in a more flexed elbow [15]. Anatomical landmarks were located as previously described [4].

Blood samples collection were obtained following the draw chart in Figure 1. During the experimental sessions, a catheter was maintained in the forearm vein and 10 mL for each draw were collected according to the protocol. Blood samples were maintained at +4 °C and the plasma was immediately separated (3000 rpm × 10 min, +4 °C) and stored at −80 °C until biochemical assays. During the sample collection, a single blood sample for each experimental point was withdrawn. All analyses performed on these samples were performed twice in triplicate. Inter- and intra-assays were performed to assess precision within and between assays.

### 2.7. EMG Data Analysis

EMG signals were recorded simultaneously to mechanical data. All data collected were analyzed off-line (OTBiolab, OTBioelettronica, Torino, Italy). The mean fibers conduction velocity (MFCV) estimated from the EMG signals was the parameter of interest. For the MVIC and for each set of the FV task, the trial chosen for EMG analysis was selected based on maximal torque. Maximal MFCV values were estimated from the two double differentials by means of the cross-correlation technique over non-overlapped adjacent epochs of 125 ms as previously described [12]. %MFCV and %torque were normalized to the maximal value of MVIC attained at BASELINE.

### 2.8. Biochemical Analyses

All chemical reagents, unless specified otherwise, were purchased from Sigma-Aldrich Chemical (St. Louis, MO, USA). Plasma CK activity was determined spectrophotometrically, according to manufactory recommendations, by a manual procedure using a commercial test kit (Greiner Diagnostic GmbH, Bahlingen-Gremany). Briefly, 50 µL of plasma was incubated in Hexokinase-Glucose 6 Phosphate-G6P Dehydrogenase buffer for 3 min and then NADPH production was followed at 340 nm for a further 3 min. Plasma lactate dehydrogenase (LDH) activity was determined spectrophotometrically by quantifying the reduction of NAD+ (measured at 340 nm) at 30°C in an assay mixture containing 0.2 M Tris-HCl (pH 7.6), 7 mM oxidized NAD+, and 55 mM lactate with a 20-μL sample. Millimolar extinction coefficient ε340 = 6.22. One unit of enzymatic activity was defined as the amount of enzyme that forms 1 μmol of product per minute.

### 2.9. Reliability

Previous test–retest reliability from our laboratory for torque and MFCV calculated from strength tests performed 2 to 10 days apart, indicated that the intraclass correlation coefficients (ICC) ranged from 0.89 to 0.96 and 0.74 to 0.93, respectively, with no significant (*p* > 0.05) differences between mean test vs. retest values.

### 2.10. Statistical Analysis

For the MVIC test, differences in the dependent variables were assessed by a two-way repeated measure analysis of variance (rANOVA) for the 2 treatments (Q, PLA) in the 8 time points (BASELINE, PRE, POST, 24 h, 48 h, 72 h, 96 h, 7 d). For the FV task, a three-way rANOVA (treatment (Q, PLA) × time (BASELINE, PRE, POST, 24 h, 48 h, 72 h, 96 h, 7 d) × 5 angular velocity (0°/s, 30°/s, 60°/s, 120°/s, 240°/s)) was performed to evaluate modifications in the variables of interest. The Greenhouse–Geisser adjustment was implemented in the event of a violation of the sphericity assumption. In case of significant interactions, a one-way rANOVA was used with a subsequent follow-up Bonferroni test performed in case of significant simple main effects. Values are presented as mean ± SD and the significance level of *p* < 0.05 was used. Statistical analyses were performed with SPSS software (Statistics v 24, IMB SPSS Inc., Chicago, IL, USA). A sample size between 12 and 16 participants was calculated a priori to provide an adequate power (α = 0.05, β = 0.80 or greater; G*Power v 3.1.9.2).

## 3. Results

### 3.1. MVIC

In order to assess that a carryover effect was not present, a T-test was performed on the data of the primary outcome. More specifically, MVIC’s torque values recorded after treatments Q and PLA were not statistically different in Q/PLA and PLA/Q subgroups of participants (*p* > 0.05).

**Figure 2:**
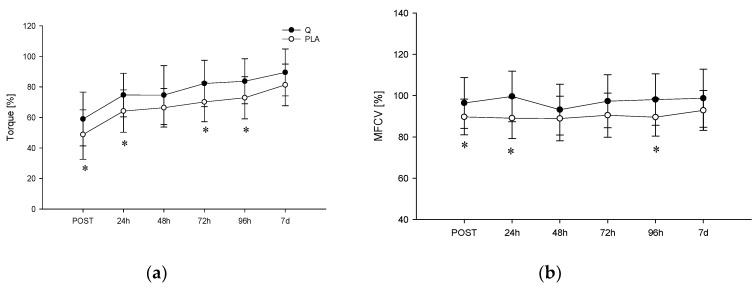
Torque (**a**) and mean fibers conduction velocity (MFCV) (**b**) of the maximal voluntary isometric contraction (MVIC) test in quercetin (Q) and placebo (PLA) before, after the eccentric exercise-induced muscle damage (EEIMD) and during the recovery. Data are expressed as % MVIC_BASE_; mean ± SD; * ≠ from Q *p* < 0.05.

Figure 2 shows the torque and MFCV values (expressed as a percentage of MVIC_BASE_) obtained during the MVIC tests. Significant effects of time and treatment were found for MFCV (*p* < 0.001 and *p* = 0.005, respectively) and torque (*p* < 0.001 and *p* = 0.007, respectively). Post hoc comparisons showed a significant difference between torque values after the eccentric protocol in Q e PLA (MVIC_POST_, *p* = 0.035; MVIC_24h,_ *p* = 0.015; MVIC_72h,_ *p* = 0.002; MVIC_96h,_ *p* = 0.026). Concerning the MFCV, significantly different values were obtained for PLA and Q conditions in MVIC_POST_ (*p* = 0.034), MVIC_24h,_ (*p* = 0.019) and MVIC_96h,_ (*p* = 0.048), which indicates a significant MFCV decrease after the eccentric protocol when participants consumed PLA.

### 3.2. FV Task

**Figure 3:**
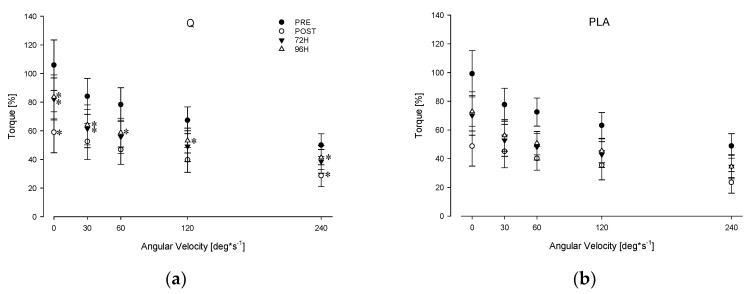
Force–velocity (FV) relationship in Q (**a**) and PLA (**b**) conditions before (PRE) and after the EEIMD (POST, 72 h, 96 h). Mean ± SD * ≠ from PLA *p* < 0.05.

Table 1 and Figure 3 show the torque and MFCV values (expressed as a percentage of MVIC_BASE_) obtained during the FV relationship task in Q and PLA conditions. Significant main effects of angular velocity, time and treatment were found for both torque (*p* < 0.001, *p* = 0.020, *p* = 0.038, respectively) and MFCV (*p* < 0.001, *p* = 0.026, *p* = 0.032, respectively). Furthermore, a significant angular velocity, treatment and time and interaction were found for torque (*p* = 0.040) and MFCV (*p* = 0.028). In particular, post hoc comparisons showed significant lower torque values POST the eccentric protocol (*p* = 0.046 at 240°/s) and after 48 h (*p* = 0.025 at 60°/s) when participants consumed PLA compared to Q. Torque values remained also lower in PLA with respect to Q at 30, 60 and 120°/s after 72 h, at all angular velocities after 96 h and at 120 and 240°/s after 7 days from the eccentric protocol (*p* < 0.05)(Figure 3).

Concerning the MFCV, post hoc comparisons showed a significantly (*p* < 0.01 on average) higher MFCV decay in POST, 24 h, 72 h, 96 h, 7 d when participants consumed PLA compared to Q at the highest angular velocities (Table 1).

### 3.3. Other EEIMD Indices

**Figure 4:**
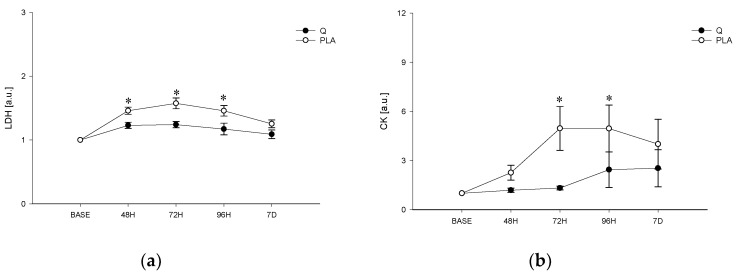
Lactate-dehydrogenase (LDH) (**a**) and creatine kinase (CK) (**b**) in Q and PLA before, after the EEIMD and during the recovery. Mean ± SD; * ≠ from Q *p* < 0.05.

Table 2 and Figure 4 show mean values of elbow angle, arm circumference, VAS, LDH and CK. In particular, a main effect of time was found for all the parameters.

Specifically, the arm circumference was significantly (*p* < 0.001) greater after the muscle damage (POST) in Q and PLA with respect to BASELINE. In addition, concerning the VAS, only a main effect of time was found but when volunteers consumed PLA and the mean values referred by the participants after the eccentric protocol (POST) were significantly higher than BASELINE (*p* = 0.017).

In POST, the elbow angle was significantly (*p* < 0.001) lower both in Q and PLA with respect to BASELINE and PRE. Post hoc comparison implemented for the time x treatment interaction, showed also that the elbow angle was lower in PLA with respect to Q (*p* = 0.034) only in POST (Table 2). A lower elbow angle indicates a more flexed arm which is in line with a rise in passive tension induced by the muscle damage.

Figure 4 shows the mean values of LDH and CK. Main effects of time and treatment and a time x treatment interaction were found. In particular, we found significantly greater levels of LDH in PLA with respect to Q 24 h, 48 h, 72 h and 96 h after the EEIMD (*p* < 0.001 at any time point). Concerning CK, mean values were different in Q and PLA only after 48 h and 72 h (*p* = 0.035 and *p* = 0.021, respectively).

## 4. Discussion

This study has shown that 14-day Q supplementation not only attenuates the magnitude of damage resulting from eccentric exercise, but also ameliorates the time course of symptoms associated with the inflammatory response of the secondary damage and accelerates the recovery of neuromuscular function.

As expected, the eccentric exercise was able to induce significant decrements in maximal isometric torque and in the force–velocity relationship in the elbow flexors muscle of the participants. In particular, the isometric strength loss recorded after the eccentric protocol (MVIC_POST_) was −51.2% with respect to baseline in PLA and −41.0% when subjects consumed Q.

The extent of muscle damage has been typically assessed by indirect markers and, among them, reduced muscle strength is considered the most appropriate [16]. The mechanical stress of the eccentric exercise, more than metabolic factors, has been shown to be the more likely candidate responsible for the primary damage [1,17]. The inhomogeneous lengthening of sarcomeres, in fact, leads to disruption of contractile and non-contractile apparatus followed by membrane damage and excitation–contraction coupling dysfunction [18]. The subsequent massive movement of Ca^2+^ into the cytoplasm causes the degradation of structural protein and apoptosis or necrosis [19]. Neutrophils activated by proteolysis, triggers the production of pro-inflammatory cytokines responsible for the secondary damage process and tissue degradation [20,21]. It has been known that the inflammatory response after muscle damage involves also the formation of reactive oxygen species (ROS) [22]. High level of ROS reacting with lipids, proteins and DNA cause their oxidative modification, and the loss of their function [23]. The antioxidative capacity of quercetin has been demonstrated in healthy subjects [24,25] and in animal models [26]. Quercetin also prevents the increase in ROS production and lipid oxidative damage induced by intense exercise [27]. Hence it is possible to speculate that quercetin can possibly improve stiffness and joint mobility as revealed by our findings on joint angle by contrasting the increase in ROS production and therefore limiting their potential harmful effect on cells. All these factors could explain why the isometric and isokinetic strength production capacity was better preserved when subjects underwent Q supplementation and the fact that this result is more evident between 72 and 96 h after eccentric exercise, when the second phase of the damage is well established. Moreover, since the dietary intervention is unlikely to affect directly the sarcomere’s lengthening during the eccentric exercise, the less severe amount of damage immediately after the protocol may be explained by an increased membrane resistance to the same mechanical stress reflected by the already reported Q-mediated reduced lipid peroxidation, better redox status and sustained muscle functions [3,28].

Previous studies on the use of dietary polyphenols in managing EEIMD, however, failed to find an attenuation of the indices of muscle damage. The discrepancy with respect to the findings from the present study can be attributed to many factors, such as the different protocols used to induce muscle damage and the different compositions of the dietary interventions, which makes a comparison very difficult. Among the studies that have used protocols comparable to that of the present investigation, O’Fallon and colleagues have reported an ineffectiveness of Q to improve muscle function and reduce inflammation or pain [9]. One of the possible reasons for this inconsistency can be ascribed to the study design that in the above-mentioned investigation, involved two different groups of subjects randomly assigned to Q supplementation or PLA. In our study the subjects acted as their own control and underwent both interventions, an aspect which may have strengthened the results of the present investigation. Another point is that the duration of the supplementation that is longer in our study and may be more effective in producing the above-mentioned adaptations in membrane resistance. In support of this hypothesis, a previous study investigating the effects of a flavonoid-based dietary supplement on eccentric exercise injury, found less elevation in the IL-6 response and C-reactive protein after 14-day supplementation [29]. The authors suggested that their results indicated a significant blunting of the systemic inflammatory response induced by EEIMD in subjects taking the flavonoids. However, the strength loss was not measured and the evolution of recovery not followed, factors that the authors themselves indicate as needing further investigation to understand how the repair of muscle damage is dependent on inflammatory mediators. In this regard, an interesting finding of our study is that plasma and functional parameters were not significantly different in PLA and Q shortly after the muscle damage (as expected for the timing of secondary phase) but the greater elevation of CK and LDH at 48, 72 and 96 h found in PLA, was not present after 7 days. Therefore, the observations made more than 96 h later may not return a complete picture of the EEIMD recovery time course, which would seem to benefit more from the Q supplementation between 72 and 96 h after the damage. On the other hand, the neuromuscular parameters were significantly different between Q and PLA immediately post-exercise without a complete return to the baseline levels after a week.

Interestingly, concerning neuromuscular parameters, the greater loss of strength recorded in PLA after eccentric exercise was accompanied also by a reduction in the mean fiber conduction velocity. However, MFCV values were not significantly reduced in Q condition. This result suggests that type IIx fibers may take better advantage of the protective action of Q since they are the most damaged by eccentric exercise. In fact, eccentric exercise may preferentially recruit fast twitch muscle fibers and induce the recruitment of inactive motor units [30]. Moreover, lengthening combined with overload has been shown to be the most relevant stimulus to increase motor units’ firing rates and that more biceps brachii motor units are active at the same relative force after eccentric exercise [25]. This can produce an unaccustomed mechanical tension in the muscle and a greater muscle damage to these fibers. As fast-glycolytic fibers characterized by the highest MFCV, it can be assumed that their contractile function remained better preserved after the eccentric damage when subjects consumed Q as revealed by the significantly lower MFCV values obtained during the isokinetic contractions performed at the highest velocities (120 and 240°/s) in placebo conditions. It has been hypothesized that also the magnitude of oxidative stress after eccentric exercise is affected by muscle fiber-type [31]. Type II fibers, which possess fewer mitochondria and rely heavily on glycolytic metabolism, have been shown to have unique properties that potentiate mitochondrial reactive oxygen species production/emission [32]. These findings are consistent with the hypothesis that the potential selective eccentric exercise-induced damage of type II myofibers, may be attenuated by dietary compounds with antioxidant properties. Among them, Q-supplementation may represent a nutritional strategy that may favorably affect skeletal muscle redox state.

Another factor to consider in explaining the time course of neuromuscular function recovery after EEIMD, is the effects that Q can exert directly on the brain. Nutrition studies often ignored central nervous system (CNS) factors, while they could be very important. In fact, quercetin is an adenosine A_1_ receptor antagonist and may enhance exercise tolerance, motivation and reduce fatigue [33]. However, further studies are needed to determine whether this observation could be translated to benefits for athletic performance or health.

### Limitations

Among the limits of the present investigation, it is necessary to include the fact that we did not measure the bioavailability of Q during the protocol. However, in order to minimize the risk of variability, we enrolled a very homogeneous group of participants in terms of age and physical state. Moreover, short Q supplementation periods seems not to be able to promote its various biological effects [34,35] since the reported estimated rate of compound elimination is less than 24 h in humans [36]. For this reason, a maintenance of high concentrations in plasma may only be achieved with Q regular and frequent dietary consumption [37,38,39]. Therefore, we have chosen to administrate Q over a period of two weeks with a twice-daily intake of Q (500 mg every 12 h) based on dietary availability [38] and according to bioavailability testing already reported in humans [37,38,39].

Another limit of this study is the potential carryover effects. The crossover design, in fact, yields a more efficient comparison of treatments than a parallel design since subjects are on their own controls and requires a smaller sample size than a parallel design, but achieves the same level of statistical power and precision. In the present study, separate baselines were assessed and treatments were spaced by a wash-out period long enough to minimize this possibility.

## 5. Conclusions

In conclusion, the results from the present study, besides extending previous evidence of the effectiveness of Q supplementation in mitigating the extent of muscle damage induced by eccentric exercise, provide new insights into the time course of symptoms associated with the recovery of neuromuscular function after EEIMD. Quercetin has the potential to represent a nutritional strategy to ameliorate skeletal muscle damage, not only for the impact on redox process but also for factors acting at the CNS level.

## Figures and Tables

**Table 1:** MFCV in Q and PLA conditions before, after the EEIMD and during the recovery at the different angular velocities of the FV task.

		MFCV (%)			
		30°/s	60°/s	120°/s	240°/s
PRE	Q	100.4 ± 5.3	101.5 ± 4.9	101.7 ± 5.1	102.5 ± 6.2
	PLA	100.9 ± 7.4	101.0 ± 4.8	101.1 ± 4.6	101.2 ± 5.9
POST	Q	97.1 ± 7.2	97.2 ± 8.0	97.2 ± 7.4	98.3 ± 7.4
	PLA	96.2 ± 9.2	96.6 ± 7.3	90.2 ± 9.1 *	87.8 ± 7.7 *
24 h	Q	95.9 ± 9.5	96.0 ± 9.0	96.7 ± 8.0	97.2 ± 8.1
	PLA	96.2 ± 5.4	97.2 ± 5.7	89.8 ± 7.7 *	86.6 ± 8.5 *
48 h	Q	98.2 ± 9.9	98.9 ± 8.1	98.8 ± 7.5	99.1 ± 7.8
	PLA	97.6 ± 6.1	97.6 ± 4.4	91.8 ± 5.0 *	89.1 ± 7.9 *
72 h	Q	99.5 ± 7.8	100.0 ± 7.3	99.8 ± 6.3	100.9 ± 6.6
	PLA	98.6 ± 3.6	97.2 ± 4.3	91.4 ± 5.3	87.4 ± 6.0 *
96 h	Q	99.6 ± 7.1	100.2 ± 6.6	100.7 ± 5.4	100.6 ± 5.5
	PLA	97.0 ± 4.8	97.6 ± 4.1	92.3 ± 4.8 *	88.7 ± 6.4 *
7 d	Q	100.7 ± 7.8	101.2 ± 6.4	101.1 ± 5.9	102.0 ± 6.2
	PLA	99.3 ± 4.6	100.2 ± 3.4	93.6 ± 4.4 *	90.8 ± 5.9 *

Data are expressed as % MFCV_BASE_; mean ± SD; * ≠ from Q *p* < 0.05. MFCV: mean fibers conduction velocity; Q: quercetin; PLA: placebo; PRE: pre-test; POST: post-test; EEIMD: eccentric exercise-induced muscle damage; FV: Force–velocity

**Table 2:** Arm circumference, visual analogue scale (VAS) of perceived pain and elbow angle in Q and PLA before, after the EEIMD and during the recovery.

		Arm Circumference	VAS	Elbow Angle
		cm	cm	degrees
BASELINE	Q	31.1 ± 0.7	0	175.3 ± 5.0
	PLA	30.6 ± 0.7	0	175.0 ± 6.3
PRE	Q	31.1 ± 0.7	0	174.3 ± 5.1
	PLA	30.7 ± 0.8	0	175.6 ± 7.3
POST	Q	32.2 ± 0.7	0.4 ± 0.2	164.7 ± 6.9
	PLA	32.2 ± 0.8	1.2 ± 0.4	158.8 ± 14.4 *
24 h	Q	31.7 ± 0.6	1.7 ± 0.3	163.1 ± 8.5
	PLA	31.7 ± 0.8	2.8 ± 0.5	158.8 ± 13.2
48 h	Q	31.7 ± 0.7	2.8 ± 0.5	158.1 ± 9.8
	PLA	31.6 ± 0.8	3.0 ± 0.5	159.1 ± 12.8
72 h	Q	31.4 ± 0.7	2.5 ± 0.6	160.0 ± 12.1
	PLA	32.0 ± 0.8	3.3 ± 0.6	155.9 ± 15.6
96 h	Q	31.7 ± 0.7	2.8 ± 0.5	164.4 ± 11.8
	PLA	31.6 ± 0.8	3.0 ± 0.5	157.8 ± 12.9
7 d	Q	31.4 ± 0.7	2.5 ± 0.6	169.7 ± 9.7
	PLA	32.0 ± 0.8	3.3 ± 0.6	168.1 ± 13.3

Mean ± SD; * ≠ from Q *p* < 0.05. Q: quercetin; PLA: placebo; PRE: pre-test; POST: post-test.
